# Authors’ Reply: “Adolescent Cocreation in Digital Health: From Passive Subjects to Active Stakeholders”

**DOI:** 10.2196/71897

**Published:** 2025-02-20

**Authors:** Callum C Lewis, Melody Taba, Tiffany B Allen, Patrina H.Y Caldwell, S Rachel Skinner, Melissa Kang, Hamish Henderson, Liam Bray, Madeleine Borthwick, Philippa Collin, Kirsten McCaffery, Karen M Scott

**Affiliations:** 1 Specialty of Child and Adolescent Health Faculty of Medicine and Health University of Sydney Sydney Australia; 2 Sydney Health Literacy Lab, School of Public Health Faculty of Medicine and Health University of Sydney Sydney Australia; 3 Children's Hospital at Westmead Sydney Australia; 4 General Practice Clinical School Faculty of Medicine and Health University of Sydney Sydney Australia; 5 Sydney School of Architecture, Design and Planning University of Sydney Sydney Australia; 6 Institute for Culture and Society Western Sydney University Richmond Australia; 7 Education Office, Sydney Medical School Faculty of Medicine and Health University of Sydney Sydney Australia

**Keywords:** adolescent health, digital health literacy, adolescents, online health information, co-design, health education, eHealth literacy, social media

The author of “Adolescent Cocreation in Digital Health: From Passive Subjects to Active Stakeholders” [1] highlights the importance of actively involving adolescents in research about adolescent health. Our co-design with adolescents, outlined in “Developing an Educational Resource Aimed at Improving Adolescent Digital Health Literacy: Using Co-Design as Research Methodology” [2], was characterized as a “pivotal intervention” because we prioritized the youth voice in its design process.

The role played by adolescents in our research was central to our development of a digital educational resource that would help adolescents improve their digital health literacy. We valued the involvement of a diverse group of adolescents as research partners in the co-design, to ensure the resource was engaging and aligned with the educational needs of a broad range of adolescents. Thus, our adolescent research partners’ insights and recommended designs were an integral part of the educational resource, which was created for use on handheld devices, based around quizzes and serious games, and featured short videos and still images that are typical of social media platforms that are popular with adolescents.

Our adolescent research partners who rejoined us for beta testing were pleasantly surprised to see that the educational resource was developed based on their design ideas, and they were pleased to have been involved and heard in the project. This highlighted the valuable combination of inputs from these diverse adolescent research partners with the equally diverse research team members, all of whom brought unique perspectives to the design. In a separate, follow-up evaluation, we found that the educational resource was effective at improving the digital health literacy of another group of adolescents, who felt that the content was engaging and met their age group’s educational needs.

Social-cognitive theory’s concept of self-efficacy (confidence in one’s abilities) was foundational in our co-design approach and the educational resource [3]. To enhance users’ self-efficacy and learning through co-design, we ensured the resource was appropriate to adolescents’ attitudes and behavior and embedded in their social context.

A participatory research approach is crucial when conducting research in adolescent health with adolescents [4]. There is increasing recognition of adolescence as an important life phase, involving multiple physical and psychosocial changes that influence health behavior and health outcomes into adulthood [5]. Nevertheless, adolescents are not a homogenous group: adolescence ranges in age from 10-25 years and involves broad differences in sociodemographic background, interests, abilities, and gender and sexual identities. When conducting participatory research with adolescents, it is important to collaborate with a group that includes members from all subpopulations of interest who have a stake in the project’s outcomes [6].

For participatory research to be maximally effective, adolescents should be involved in the actual research design. This has implications for research planning, with time required for recruiting adolescents to the research team and research training. Acknowledgement and remuneration are required in appreciation of the adolescents’ lived experience expertise and time dedicated to research training and design. Adolescents’ contributions also need to be in ethics applications to human research ethics committees. When seeking research funding, budgetary provisions need to be available for this preparatory phase of participatory research [7].
